# SmbHLH37 Functions Antagonistically With SmMYC2 in Regulating Jasmonate-Mediated Biosynthesis of Phenolic Acids in *Salvia miltiorrhiza*

**DOI:** 10.3389/fpls.2018.01720

**Published:** 2018-11-22

**Authors:** Tangzhi Du, Junfeng Niu, Jiao Su, Shasha Li, Xiaorong Guo, Lin Li, Xiaoyan Cao, Jiefang Kang

**Affiliations:** National Engineering Laboratory for Resource Development of Endangered Crude Drugs in Northwest of China, Key Laboratory of the Ministry of Education for Medicinal Resources and Natural Pharmaceutical Chemistry, Shaanxi Normal University, Xi’an, China

**Keywords:** bHLH, JA signaling, JAZ, secondary metabolites, *SmTAT1*, *SmPAL1*

## Abstract

Jasmonates (JAs) are integral to various defense responses and induce biosynthesis of many secondary metabolites. MYC2, a basic helix-loop-helix (bHLH) transcription factor (TF), acts as a transcriptional activator of JA signaling. MYC2 is repressed by the JASMONATE ZIM-domain (JAZ) proteins in the absence of JA, but de-repressed by the protein complex SCF^COI1^ on perception of JA. We previously reported that overexpression of *SmMYC2* promotes the production of salvianolic acid B (Sal B) in *Salvia miltiorrhiza.* However, the responsible molecular mechanism is unclear. Here, we showed that SmMYC2 binds to and activates the promoters of its target genes *SmTAT1*, *SmPAL1*, and *SmCYP98A14* to activate Sal B accumulations. *SmbHLH37*, a novel bHLH gene significantly up-regulated by constitutive expression of *SmMYC2*, was isolated from *S. miltiorrhiza* for detailed functional characterization. SmbHLH37 forms a homodimer and interacts with SmJAZ3/8. Overexpression of *SmbHLH37* substantially decreased yields of Sal B. SmbHLH37 binds to the promoters of its target genes *SmTAT1* and *SmPAL1* and blocks their expression to suppress the pathway for Sal B biosynthesis. These results indicate that SmbHLH37 negatively regulates JA signaling and functions antagonistically with SmMYC2 in regulating Sal B biosynthesis in *S. miltiorrhiza*.

## Introduction

*Salvia miltiorrhiza* Bunge, a well-known member of the Labiatae family, is considered a model medicinal plant (Guo et al., 2014). Its dry roots and rhizomes (called ‘danshen’ in Chinese) are widely applied in the treatment of various cerebrovascular and cardiovascular diseases (Han et al., 2008; Zeng et al., 2013; Su et al., 2015). The major bioactive components of *S. miltiorrhiza* are classified as water-soluble phenolic acids, including salvianolic acid B (Sal B) and rosmarinic acid (RA); and lipid-soluble tanshinones such as cryptotanshinone and tanshinone IIA (Ma et al., 2013; Ma et al., 2015). Phenolic acids are attracting increased attention because of their marked pharmacological activities coupled with their traditional use as herbs steeped in boiling water in China. Among these phenolic acids, Sal B is predominant and is regarded for its antioxidant properties and scavenging of free radicals (Zhao et al., 2008). It offers protection against fibrosis, tumor development, aging, and cardiovascular/cerebrovascular diseases (Zhao et al., 2008; Tsai et al., 2010).

The biosynthetic pathway of Sal B consists of a phenylalanine-derived pathway and tyrosine-derived pathway (Di et al., 2013; Ma et al., 2013; Wang et al., 2015). In view of the economic value and clinical demand for this active ingredient, biological approaches have been taken to augment its synthesis, including the engineering of genes in the biosynthetic pathway and ectopic expression of transcription factors (TFs) (Zhang et al., 2010, 2014; Wang et al., 2013; Zhou et al., 2016; Yang et al., 2017). For example, AtPAP1 from *Arabidopsis thaliana* is a transcriptional activator of phenolic acid biosynthesis in *S. miltiorrhiza* (Zhang et al., 2010, 2014). Heterologous expression of two TFs, Delila (DEL) and Rosea1 (ROS1) from *Antirrhinum majus*, significantly elevates the production of Sal B in *S. miltiorrhiza* (Wang et al., 2013). In addition, exogenous application of methyl jasmonate (MeJA) triggers an extensive transcriptional reprogramming of metabolism and dramatically increases Sal B biosynthesis in that species (Ge et al., 2015).

Jasmonates (JAs) play crucial roles in plant responses to various stimuli and induce biosynthesis of many secondary metabolites (Browse, 2009; Zhou and Memelink, 2016). The Jasmonate ZIM-domain (JAZ) proteins function as negative regulators to repress diverse JA responses (Chini et al., 2007; Thines et al., 2007; Seo et al., 2011; Song et al., 2011). Jasmonoyl-_L_-isoleucine (JA-Ile), the active form of JA, promotes the degradation of Jasmonate ZIM-domain (JAZ) proteins via the 26S proteasome system (Farmer, 2007; Sheard et al., 2010). This is followed by de-repression of MYC2, a basic helix-loop-helix (bHLH) TF that has a central role in JA signaling, resulting in transcriptional activation of downstream target genes (Lorenzo et al., 2004; Chico et al., 2008; Katsir et al., 2008). Nine JAZ genes have been cloned from *S. miltiorrhiza* and some have been functionally verified as negative regulators of active ingredients in this species. For example, overexpression of *SmJAZ8* de-regulates the yields of salvianolic acids and tanshinones in MeJA-induced transgenic hairy roots (Ge et al., 2015; Pei et al., 2018). Both SmJAZ3 and SmJAZ9 act as repressive transcriptional regulators in the biosynthesis of tanshinones (Shi et al., 2016). However, SmMYC2a and SmMYC2b, two orthologs of MYC2, interact with SmJAZs and positively regulate the biosynthesis of tanshinones and Sal B in *S. miltiorrhiza* hairy roots (Zhou et al., 2016).

The bHLH proteins, one of the largest TF families in plants, modulate various physiological or morphological events, including different branches of the flavonoid pathway (Carretero-Paulet et al., 2010; Hichri et al., 2011). The bHLH family consists of an N-terminal stretch of basic amino acid residues responsible for DNA binding and an HLH domain to form homo- or heterodimers (Goossens et al., 2017), which bind E-box sequences (CANNTG), such as the G-box (CACGTG), in the promoter of their target genes (Ezer et al., 2017). The bHLHs are monophyletic and constitute 26 subfamilies characterized by the presence of highly conserved short amino acid motifs (Pires and Dolan, 2010). MYC2, a member of bHLH subgroup IIIe, positively regulates secondary metabolism during JA signaling in a species-specific manner (Dombrecht et al., 2007; Todd et al., 2010; Zhang H. et al., 2011). JA-ASSOCIATED MYC2-LIKE1 (JAM1), JAM2, and JAM3 (bHLH17, -13, and -3, respectively) belong to the bHLH IIId subfamily in *A. thaliana*. Each contains a domain that can interact with JAZ proteins and negatively regulate JA responses (Fonseca et al., 2014; Sasaki-Sekimoto et al., 2014). JAM1 substantially reduces those responses, inhibiting root growth and interrupting anthocyanin accumulations and male fertility (Nakata et al., 2013). JAM2 and JAM3 have the same functions and act redundantly with JAM1 (Nakata and Ohme-Takagi, 2013). These JAMs antagonize MYC2, MYC3, and MYC4 during JA-induced leaf senescence by binding to the same target sequences of MYC-activated genes (Qi et al., 2015a).

Zhang et al. (2015) have identified 127 bHLH genes in *S. miltiorrhiza* based on genome-wide analyses. They have predicted seven *bHLH*s, including *SmbHLH37*, that are involved in tanshinone biosynthesis. However, the functions of those genes have not been characterized. We previously reported that overexpression of *SmMYC2* increases the production of phenolic acids in *S. miltiorrhiza* (Yang et al., 2017). Further investigation showed that constitutive expression of that gene significantly up-regulates transcript levels of *SmbHLH37* (Su et al., 2017). Multiple alignments of the SmbHLH37 protein sequence with AtbHLHs from *Arabidopsis* have indicated that SmbHLH37 is most closely correlated with AtbHLH3 (JAM3), both of which belong to the IIId subfamily (Su et al., 2017). In the present study, we identified SmbHLH37 as a new target of JAZ proteins. We then conducted overexpression experiments to explore the function of *SmbHLH37* in *S. miltiorrhiza*. Transgenic overexpressing (OE) plants showed significantly lower accumulations of Sal B. We concluded that SmbHLH37 antagonizes the previously reported transcription activator SmMYC2 in controlling salvianolic acid biosynthesis in *S. miltiorrhiza* by binding to their downstream target sequences. Coordinated regulation of Sal B by this transcription repressor and activator provides clues about the previously unknown complex mechanism for directing the production of secondary metabolites.

## Materials and Methods

### Experimental Materials

Sterile *Salvia miltiorrhiza* plantlets were cultured on a Murashige and Skoog basal medium, as described previously (Yan and Wang, 2007). All chemicals were obtained from Sigma Chemical Co. (St. Louis, MO, United States). Solvents were of high-performance liquid chromatography (HPLC) grade. Standards of RA, Sal B, and JA were purchased from the National Institute for the Control of Pharmaceutical and Biological Products (Beijing, China). All were prepared as stock solutions in methanol and stored in the dark at -18°C. Primer pairs are listed in Supplementary Tables 1, 2.

### Construction of Plant Expression Vectors and Plant Transformation

To construct the *SmbHLH37* overexpression vector, we amplified the full-length open reading frame (ORF) of *SmbHLH37* (GenBank Accession Number KP257470.1) with primers GV*SmbHLH37*-F/R, which introduced attB sites, and subsequently re-combined it into the pDONR207 vector (BP reaction Gateway^®^) according to the protocol from the Gateway manufacturer (Invitrogen, United States). The ENTRY vector pDONR207-*SmbHLH37* was sequenced and inserted into the pEarleyGate 202 vector (Earley et al., 2006) by an LR reaction (Gateway^®^) to generate the pEarleyGate 202-*SmbHLH37* overexpression vector. *Agrobacterium*-mediated gene transfer was performed based on protocols established in our laboratory (Yan and Wang, 2007).

### Molecular Characterization of Transgenic Plantlets

To evaluate whether the OE box had been integrated into the transgenic plant genome, we amplified the *CaMV35S* promoter from isolated genomic DNA, using previously published protocols (Yang et al., 2017).

Total RNA from the roots of *S. miltiorrhiza* transgenic lines was extracted and converted into cDNA. Gene expression was monitored via real-time quantitative PCR (RT-qPCR), with housekeeping gene *SmUbiquitin* serving as an internal reference. Quantitative reactions were performed on a LightCycler^®^ 96 real-time PCR detection system (Roche, Switzerland), using SYBR Premix Ex TaqTM (Takara, Beijing, China). The reaction mixture contained 10 μl of 2 × SYBR Premix Ex Taq II, 0.5 μM each of sense and antisense primers, 20 ng of first-strand cDNA, ddH_2_O up to 20 μl. Initial thermal-cycling at 95°C for 30 s was followed by 45 cycles of 95°C for 10 s and 60°C for 30 s. All experiments were performed on three independent biological experiments with each including three technical replicates. Relative expression was analyzed according to the 2^-ΔΔCt^ method compared with the WT (Livak and Schmittgen, 2001). Statistical significance was calculated using the by two-tailed Student’s *t*-test.

Based on the transcript levels of *SmbHLH37*, we conducted RT-qPCR analysis to determine the expression levels of key enzyme genes for the biosynthetic pathways of Sal B, JA, and anthocyanin.

### Determination of Anthocyanin Concentrations

Extraction and quantification of anthocyanins was performed in accordance with the protocols of Mano et al. (2007), with minor modifications. 20 mg samples of powder from transgenic or wild type (WT) plants were extracted with 1 mL of acidic methanol [1% (v/v) HCl] for 1 h at 20°C, with moderate shaking (100 rpm). After centrifugation (12000 rpm, room temperature, 5 min), 0.7 mL of the supernatant was added to 0.7 mL of chloroform. Absorption of the extracts at wavelengths of 530 and 657 nm was determined photometrically (DU 640 Spectrophotometer, Beckman Instruments). Quantitation of anthocyanins was performed using the following equation: Q (anthocyanins) = (A530–0.25 × A657) × M^-1^, where Q (anthocyanins) is the concentration of anthocyanins, A530 and A657 are the absorptions at the wavelengths indicated, and M is the dry weight (in grams) of the plant tissue used for extraction.

### Determination of Phenolics and JA Concentrations by LC/MS Analysis

Roots were collected from 2-month-old transgenic plantlets and air-dried at 20 ± 2°C. The phenolic compounds were extracted and determined as described by Li et al. (2018).

To determine the concentration of JA, we extracted JA using a modified protocol as described (Yang et al., 2012). Approximately 0.1-g root samples were homogenized and added to 10 mL of cold extraction buffer (acetone: 50 mM citric acid, 7:3, v/v). After this mixture was vortexed and then left to stand 30 min at 4°C, 10 mL of ethyl acetate was added before vortexing again. Following centrifugation at 5000 g for 10 min at 4°C, the supernatants were transferred to new 50-mL tubes and evaporated to dryness in a freeze dryer. The residue of each sample was re-suspended in 1 mL of 80% methanol (v/v) and sonicated for 10 min, then passed through a 0.22-μm organic filter. The extracts were loaded onto an Agela Cleanert SPE-NH2 (500 mg/6 mL); sonication and filtration steps were repeated. The combined supernatants were used for JA detection.

We determined the concentrations of JA in the plant samples by LC-QQQ-MS. Briefly, analyses were conducted using an Agilent 1260 HPLC system coupled to an Agilent 6460 QQQ LC-MS system equipped with a dual electrospray ion source operated in the negative mode. The extracts were separated on a Welch Ultimate XB-C18 column (2.1 × 150 mm, 3 μm). The chromatographic separation was performed over an 8-min analysis time, using a linear gradient of 85% to 50% A (0–6 min), 50% to 0% A (6–7 min), and 0% to 0% A (7–8 min). The flow rate of the gradient mobile phase was 0.4 mL/min, and the column temperature was 30°. Conditions for mass spectrometry included a drying gas temperature of 300°C, drying gas flow of 10 L/min, nebulizer pressure of 45 psi, ion spray voltage of 3500 V, and sheath gas of 11 L/min, at a temperature of 350°C. Retention time was 6.9 min for JA. The precursor/product ion of JA was 209.1 > 59.1. The concentrations were quantified based on standard curves prepared with authentic reference standards.

### Bimolecular Fluorescent Complementation (BiFC)

The ORFs of *SmJAZ1*/*3*/*8* and *SmMYC2* without the termination codon were individually cloned into the pDONR207 vector through Gateway reactions and re-combined into the pEarleyGate202-YC (YC) vector to generate YC-*SmJAZ1/3/8* and YC- *SmMYC2*. Likewise, the ORF of *SmbHLH37* without the termination codon was inserted into the YC vector or pEarleyGate201-YN (YN) to construct YC-*SmbHLH37* and YN-*SmbHLH37*. The YC and YN recombinant plasmids were mixed at equal densities before co-transformation.

The plasmids were transiently transformed into onion epidermis cells by particle bombardment (helium pressure, 1100 psi) with the PDS-1000/He system (Bio-Rad, CA, United States). After 24 h of incubation, those cells were stained with DAPI (Vector Labs, CA, United States) for 20 min and then observed using a Leica DM6000B microscope (Leica, Germany) with an excitation wavelength of 475 nm.

### Yeast Two-Hybrid (Y2H) Assays

The full-length coding sequence of *SmbHLH37* was cloned into the pGADT7 or pGBKT7 vector, while those of *SmJAZ1/3/8* and *SmMYC2* were cloned into the pGADT7 vector. To test potential auto-activation of the prey, vectors of pGBKT7-*SmbHLH37* and pGADT7 were co-transformed. Empty vectors of pGADT7 and pGBKT7 were also co-transformed as a negative control. The two types of recombinant vectors were co-transformed into yeast strain AH109 by the PEG/LiAC method (Zhou and Memelink, 2016). Interaction assays were performed according to manufacturer’s protocol for the Matchmaker Gold Yeast Two-Hybrid System (Clontech, United States), and Y2H images were taken on Day 5 of incubation.

### Yeast One-Hybrid (Y1H) Assays

The ORFs of *SmbHLH37* and *SmMYC2* were individually amplified by PCR using primers containing *Bam*HI and *Eco*RI restriction sites. They were fused to the GAL4 activation domain in vector pGADT7-Rec2 (Clontech) to create the fusion proteins pGADT7-*SmbHLH37* and pGADT7-*SmMYC2*. The ∼798-bp, ∼1350-bp, and ∼1146-bp promoter regions of *SmPAL1*, *SmTAT1*, and *SmCYP98A14*, respectively, were amplified and cloned into pHIS2 (Clontech). These recombinant vectors were co-transformed into yeast strain Y187 according to the reported protocol (Huang et al., 2013). The transformed cells were cultured on an SD/-Leu/-Trp medium and then selected on an SD/-Leu/-Trp/-His medium supplemented with 60 mM 3-amino-1, 2, 4-triazole to examine any protein-DNA interactions.

### Assay of Transient Transcriptional Activity (TTA) in *Nicotiana benthamiana*

For assaying transient transcriptional activity, we amplified and cloned the ∼798-bp, ∼1350-bp, and ∼1146-bp promoter regions of *SmPAL1*, *SmTAT1*, and *SmCYP98A14*, respectively, into the pGreenII 0800-LUC (luciferase) vector (Hellens et al., 2005) to generate our reporter construct. The full-length coding sequences of *SmMYC2* and *SmbHLH37* were inserted into the pGreenII62-SK vector as the effector. Transient expression was monitored in *N. benthamiana* leaves according to the protocols of Sparkes et al. (2006). After 3 d of infiltration, activities of firefly LUC and renillia luciferase (REN) were measured using a dual-luciferase reporter gene assay kit (Beyotime Biotechnology, China) and a GloMax 20/20 luminometer (Promega, United States). Relative LUC activity was calculated by normalizing it against REN activity.

## Results

### SmbHLH37 Forms Homodimer and Interacts With SmJAZ3/8

**FIGURE 1:**
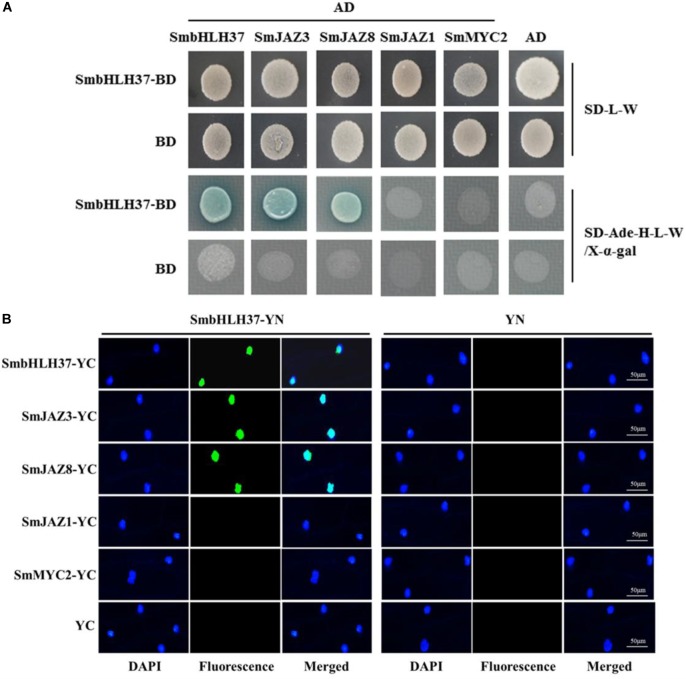
SmbHLH37 interacts with SmJAZ3, SmJAZ8, and SmbHLH37. **(A)** Yeast two-hybrid assay to detect interactions. SmJAZ1, SmJAZ3, SmJAZ8, SmMYC2, and SmbHLH37 were fused with activation domain (AD) while SmbHLH37 was simultaneously fused with DNA-binding domain (BD). Transformed yeast cells were grown on SD/-Ade/-Leu/-Trp/-His/X-α-gal media. Different rows represent individual dilutions of cells. **(B)** Bimolecular fluorescent complementation experiments in onion epidermis cells. SmJAZ1, SmJAZ3, SmJAZ8, SmMYC2, and SmbHLH37 were fused with C-terminal of fluorescin to produce SmJAZ1-YC, SmJAZ3-YC, SmJAZ8-YC, SmMYC2-YC, and SmbHLH37-YC, respectively. SmbHLH37 was fused with N-terminal of fluorescin to produce SmbHLH37-YN. Recombinant vectors were co-transformed with corresponding empty vectors as control. Nucleus was located after staining with DAPI.

*SmbHLH37* can be dramatically induced by exogenous MeJA (Zhang et al., 2015). We previously showed that SmbHLH37 is most similar to AtJAM3 (Su et al., 2017), which interacts with JAZs in *Arabidopsis* (Fonseca et al., 2014; Sasaki-Sekimoto et al., 2014). To detect whether SmbHLH37 and SmJAZs could interact with each other in *S. miltiorrhiza*, we performed BiFC and Y2H assays. Because *SmJAZ1* and *SmJAZ8* quickly respond exogenous MeJA treatment and SmJAZ3 shows highest expression in roots (Ge et al., 2015), we selected SmJAZ1/3/8 for the experiments. Our results demonstrated that SmbHLH37 interacts with SmJAZ3/8 (Figures 1A,B). Among the JAZ proteins in *S. miltiorrhiza*, SmJAZ8 has been established as being involved in repressing the biosynthesis of salvianolic acids and tanshinones (Ge et al., 2015; Pei et al., 2018). The bHLH protein usually forms a homodimer or heterodimer to develop their function (Feller et al., 2006). Our findings indicated that SmbHLH37 does form a homodimer (Figures 1A,B). Moreover, SmbHLH37 does not interact with SmMYC2.

### Overexpression of *SmbHLH37* Decreases Endogenous JA Concentrations and Affects JA Signal Pathway

To check whether the expression box had been integrated into the genome of *S. miltiorrhiza*, we performed PCR to amplify the CaMV 35S promoter. Our result showed that the transgenic plants indeed contained an expected 721-bp fragment (Supplementary Figure 1A). Real-time quantitative PCR demonstrated that expression of *SmbHLH37* was highest in Lines OE-4 and OE-7 when compared with the non-transformed WT (Supplementary Figure 1B). Therefore, we chose those two lines for further analysis.

**FIGURE 2:**
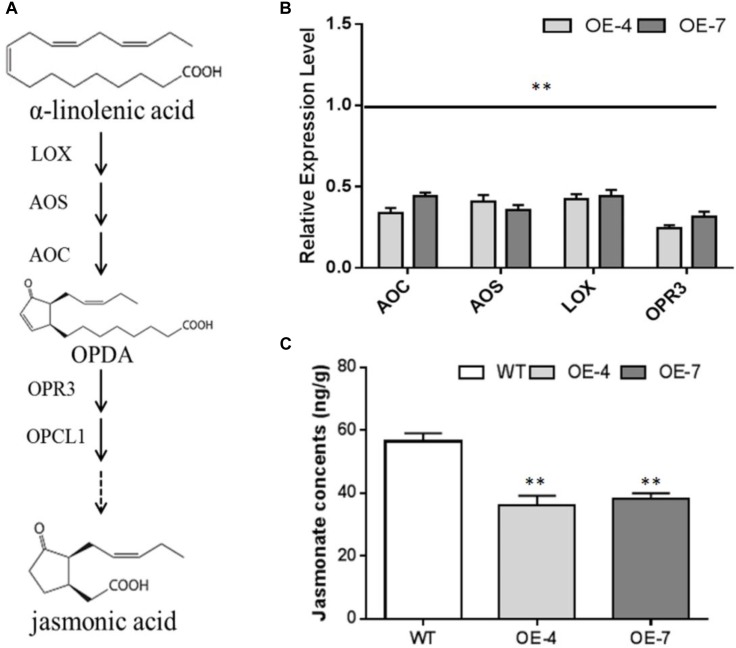
Effects of *SmbHLH37* overexpression on pathway of JA biosynthesis. **(A)** Pathway Enzymes: LOX, lipoxygenase; AOS, allene oxide synthase; AOC, allene oxide cyclase; OPR3, 12-oxophytodienoate reductase 3. **(B)** Relative expression levels of genes involved in JA biosynthesis pathway. Expression values in WT were set to ‘1’ (not shown). *LOX* (SMil_00027821-RA_Salv), *AOC* (SMil_00024799-RA_Salv), and *AOS* (SMil_00002529-RA_Salv) were retrieved from the web portal at http://www.ndctcm.org/shujukujieshao/2015-04-23/27.html. *OPR3* (KF220568.1) was retrieved from GenBank databases **(C)** Concentrations of JA in root extracts from *SmbHLH37*-overexpressing lines (OE) and wild type (WT). All data are means of 3 replicates, with error bars indicating SD; ^∗∗^; values are significantly different from WT at *p* < 0.01, by two-tailed Student’s *t*-test.

JA is derived from a-linolenic acid and the biosynthesis pathway was shown in Figure 2A. The transcript levels of genes encoding LOX (lipoxygenase), AOS (allene oxide synthase), AOC (allene oxide cyclase), and OPR3 (12-oxophytodienoic acid reductase) were significantly down-regulated in OE lines (Figure 2B). We performed LC-MS to determine the concentrations of endogenous JA in fresh root samples from OE and WT lines. The MRM chromatograms of JA were shown in Supplementary Figure 2. The results of the LC-MS analysis revealed that those JA levels were significantly decreased in OE-4 and OE-7 when compared with the control (Figure 2C). These results implied that overexpression of *SmbHLH37* lowed JA biosynthesis in *S. miltiorrhiza*.

**FIGURE 3:**
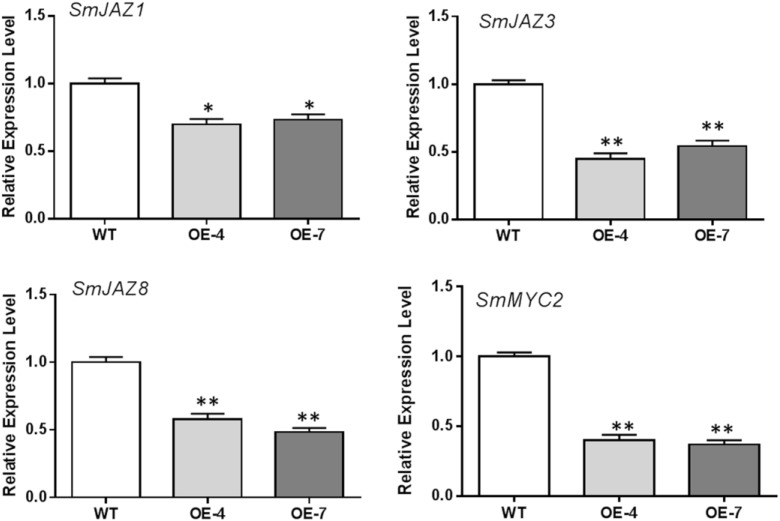
Results of RT-qPCR analysis on expression levels of *SmJAZ*s and *SmMYC2* in *SmbHLH37*-OE and WT. All data are means of 3 biological replicates, with error bars indicating SD; ^∗^ and ^∗∗^, values are significantly different from WT at *p* < 0.05 and *p* < 0.01, respectively, by two-tailed Student’s *t*-test. The following genes were retrieved from GenBank databases: *SmJAZ1* (JQ936590.1), *SmJAZ3* (KC864780.1), *SmJAZ8* (JQ936591.1), *SmMYC2* (KJ945636.1).

We further examined the transcription changes of genes encoding JAZ proteins and MYC2, core factors in the JA signaling pathway. Our RT-qPCR results showed that overexpression of *SmbHLH37* significantly decreased the transcript levels of *SmJAZ1/3/8* and *SmMYC2* (Figure 3).

### SmbHLH37 Negatively Regulates Anthocyanin Biosynthetic Pathway Through Transcriptional Cascade

**FIGURE 4:**
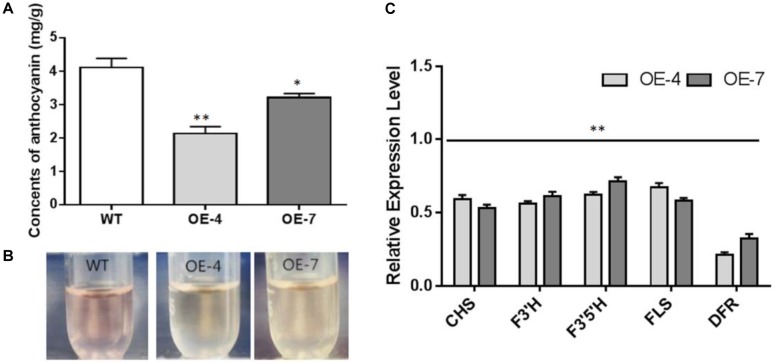
Effects of *SmbHLH37* overexpression on pathway for anthocyanin biosynthesis. **(A)** Concentrations of anthocyanin in roots of *SmbHLH37*-OE and WT. **(B)** Color of root extracts. **(C)** Relative expression levels of genes involved in pathway. CHS, chalcone synthase; F3’H, flavonoid 3’-hydroxylase; F3’5’H, flavonoid 3’5’-hydroxylase; FLS, flavonol synthase; DFR, dihydroflavonol 4-reductase. Expression values in WT were set to ‘1’ (not shown). The following genes were retrieved from GenBank databases: *CHS* (MH447681.1), *F3’H* (MH447668.1), *F3’5’H* (MH447665.1), *FLS* (MH447674.1), *DFR* (MH447664.1). All data are means of 3 biological replicates, with error bars indicating SD; ^∗^ and ^∗∗^, values are significantly different from WT at *p* < 0.05 and *p* < 0.01, respectively, by two-tailed Student’s *t*-test.

Our lab previously proved that activation of JA signaling can improve the accumulation of anthocyanin in *S. miltiorrhiza* (Ge et al., 2015). We tested whether this regulation of a transcriptional cascade by SmbHLH37 alters anthocyanin levels and found that concentrations of this pigment were significantly lower in the roots of OE-4 and OE-7 than in the WT (Figures 4A,B). We also investigated the expression profiles of genes for anthocyanin biosynthesis, e.g., *CHS* (chalcone synthase), *F3′H* (flavonoid 3′-hydroxylase), *F3′5′H* (flavonoid 3′5′-hydroxylase), *FLS* (flavonol synthase), and *DFR* (dihydroflavonol 4-reductase). All were significantly down-regulated in OE lines, with *DFR* showing the largest fold-change (Figure 4C).

### Overexpression of *SmbHLH37* Decreases Concentrations of Phenolic Acids

**FIGURE 5:**
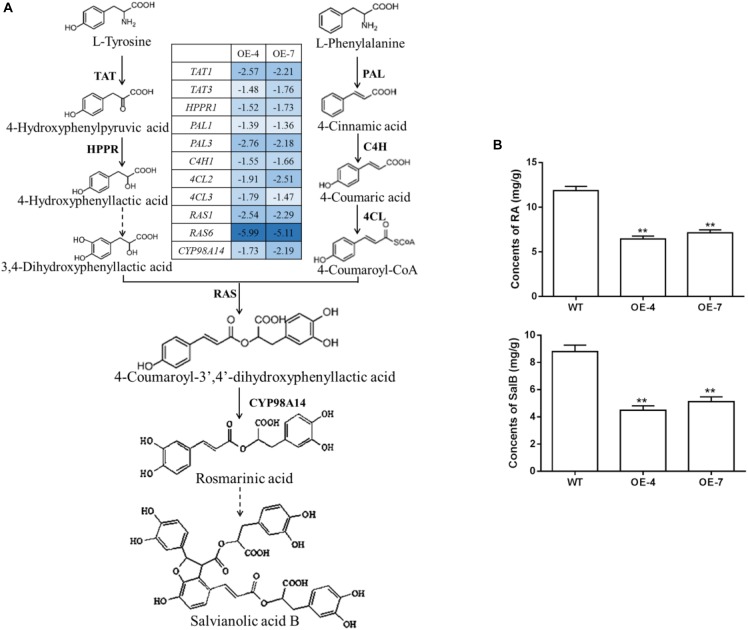
Effects of *SmbHLH37* overexpression on pathway for salvianolic acid biosynthesis. **(A)** Proposed pathway for Sal B biosynthesis and expression changes of the pathway genes in the roots of *SmbHLH37*-OE by RT-qPCR analysis. TAT, tyrosine aminotransferase; HPPR, hydroxyl phenylpyruvate reductase; PAL, phenylalanine ammonia lyase; C4H, cinnamate 4-hydroxylase; 4CL, hydroxycinnamate-CoA ligase; RAS, rosmarinic acid synthase; and CYP, cytochrome P450. Negative value in array indicates down-regulated fold change in OE lines (OE-4 and OE-7) relative to WT. The values are means of 3 biological replicates and are calculated base on the 2^-ΔΔCt^ method compared with the WT. The following genes were retrieved from GenBank databases: *TAT1* (DQ334606.1), *TAT3* (KF220555.1), *PAL1* (EF462460.1), *PAL3* (KF220569.1), *HPPR* (DQ099741.1), *C4H1* (DQ355979.1), *4CL2* (AY237164.1), *4CL3* (KF220556.1), *RAS1* (FJ906696.1), *RAS6* (KF220574.1), *CYP98A14* (HQ316179.1). **(B)** Concentrations of salvianolic acid B (Sal B) and rosmarinic acid (RA) accumulated in roots of OEs and WT, determined by LC-MS. All data are means of 3 biological replicates, with error bars indicating SD; ^∗∗^, values are significantly different from WT at *p* < 0.01, by two-tailed Student’s *t*-test.

The biosynthetic pathway of RA and Sal B included both phenylpropanoid-derived and tyrosined-derived pathway (Figure 5A). Many of the genes encoding enzymes on the pathway have been identified in *S. miltiorrhiza*, including *SmPALs*, *SmC4Hs*, *Sm4CLs*, *SmTATs*, *SmHPPRs*, *SmRASs*, and *SmCYP98A14* (Di et al., 2013; Hou et al., 2013; Wang et al., 2015). We predicted that the production of salvianolic acids would be decreased in OE lines because of the decline in JA levels. To test this, we performed LC-MS to determine the concentrations of RA and Sal B. The MRM chromatograms of RA and Sal B were shown in Supplementary Figure 3. The results of the LC-MS analysis were consistent with our expectations, i.e., the levels of RA and Sal B were significantly declined in OE lines (respective reductions of 2.0- and 1.8-fold for RA and Sal B in OE-4; 1.7- and 1.6-fold for RA and Sal B in OE-7) when compared with the WT (Figure 5). To evaluate how the expression of genes related to phenolic acid biosynthesis is influenced in transgenic lines, we monitored relative transcript levels for *SmPAL1/3*, *SmC4H1*, *Sm4CL2/3*, *SmTAT1/3*, *SmHPPR1*, *SmRAS1/6*, and *SmCYP98A14* in the WT and OE lines (Figure 5). Expression of all tested genes was significantly decreased in OE plants (*p* < 0.01). In particular, transcript levels of *RAS6* were decreased 6.0- and 5.1-fold in OE-4 and OE-7, respectively.

### SmbHLH37 Binds to and Represses Promoters of *SmTAT1* and *SmPAL1*

**FIGURE 6:**
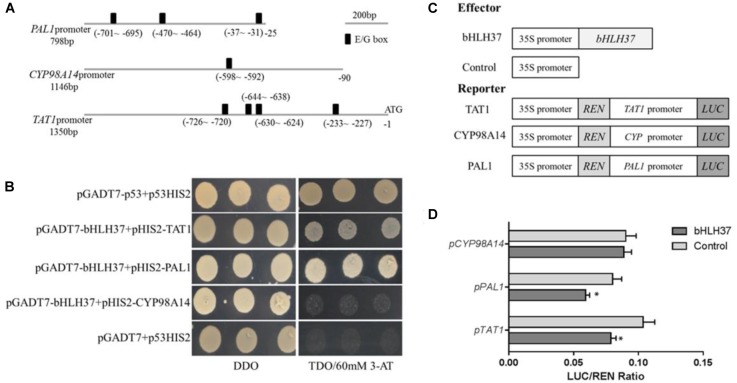
SmbHLH37 binds to and represses promoters of *SmTAT1* and *SmPAL1*. **(A)** E-box fragments of *SmTAT1*, *SmPAL1*, and *SmCYP98A14* promoters. **(B)** Yeast one-hybrid assay to detect interaction between SmbHLH37 and promoters of *SmTAT1*, *SmPAL1*, and *SmCYP98A14*. SmbHLH37 was fused to GAL4 AD. Promoter regions of *SmTAT1*, *SmPAL1* and *SmCYP98A14* were cloned into pHIS2 to construct pHIS2-*SmTAT1*, pHIS2-*SmPAL1*, and pHIS2-*SmCYP98A14*, respectively. Recombinant vectors were co-transformed into yeast strain Y187, and transformed cells were cultured on SD/-Leu/-Trp medium (DDO), then selected on SD/-Leu/-Trp/-His medium (TDO) supplemented with 60 mM 3-amino-1, 2, 4-triazole (3-AT) to examine protein-DNA interaction. The p53HIS2/pGADT7-p53 and p53HIS2/pGADT7 served as positive control and negative control, respectively. **(C)** Schematic diagram of constructs used in assays of transient transcriptional activity. **(D)** SmbHLH37 represses promoters of *SmTAT1* and *SmPAL1*. Effector SmbHLH37 was co-transformed with reporters *P_TAT1_-LUC*, *P_PAL1_-LUC*, and *P_CY P98A14_-LUC*. All data are means of 3 biological replicates, with error bars indicating SD; ^∗^, values are significantly different from WT at *p* < 0.05, by two-tailed Student’s *t*-test.

The bHLH TFs function by binding to the E/G-box of the target gene promoter. Although 29 enzyme genes have been predicted to participate in phenolic acid biosynthesis in *S. miltiorrhiza* (Wang et al., 2015), only a few have been verified as doing so, including *SmPAL1* (Song and Wang, 2011), *SmTAT1* (Xiao et al., 2011), and *SmCYP98A14* (Di et al., 2013). Each of them carries E/G-box sequences in its promoter (Figure 6A). We speculated whether SmbHLH37 is directly involved in regulating the pathway of phenolic acid biosynthesis. We conducted a Y1H experiment with promoter regions of ∼798-bp, ∼1350-bp, and ∼1146-bp length from genes *SmPAL1*, *SmTAT1*, and *SmCYP98A14*, respectively. The results shown in Figure 6B indicate that SmbHLH37 directly binds to the promoters of *SmTAT1* and *SmPAL1* rather than *SmCYP98A14* in yeast.

We then conducted an assay of transient transcriptional activity in *N. benthamiana* leaves. The promoter regions of *SmTAT1*, *SmPAL1*, and *SmCYP98A14* were fused individually with LUC to generate the reporter, and SmbHLH37, driven by the 35S promoter, was used as an effector (Figure 6C). As showed in Figure 6D, the LUC activity under promoters of *SmTAT1* and *SmPAL1* were reduced, which is due to repression of expression resulting from SmbHLH37 binding to the promoters. The same was not true for *SmCYP98A14*. Therefore, these results demonstrated that SmbHLH37 directly binds to the promoter regions of *SmTAT1* and *SmPAL1* to repress their expression.

### SmMYC2 Binds to and Activates Promoters of *SmTAT1*, *SmPAL1*, and *SmCYP98A14*

**FIGURE 7:**
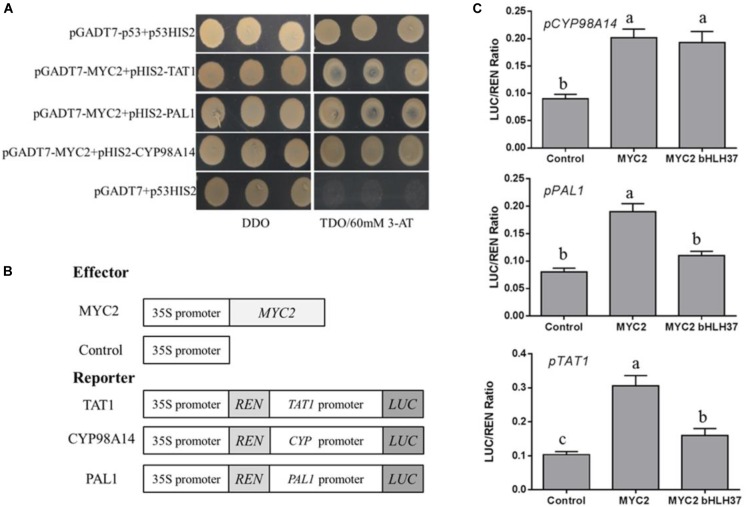
SmMYC2 binds to and activates promoters of *SmTAT1*, *SmPAL1*, and *SmCYP98A14*. **(A)** Yeast one-hybrid assay to detect interaction between SmMYC2 and promoters. SmMYC2 was fused to GAL4 AD. Promoter regions of *SmTAT1*, *SmPAL1*, and *SmCYP98A14* were cloned into pHIS2 to construct pHIS2-*SmTAT1*, pHIS2-*SmPAL1*, and pHIS2-*SmCYP98A14*, respectively. Recombinant vectors were co-transformed into yeast strain Y187 and transformed cells were cultured on SD/-Leu/-Trp medium (DDO), then selected on SD/-Leu/-Trp/-His medium (TDO) supplemented with 60 mM 3-amino-1, 2, 4-triazole (3-AT) to examine protein-DNA interaction. The p53HIS2/pGADT7-p53 and p53HIS2/pGADT7 served as positive control and negative control, respectively. **(B)** Schematic diagram of constructs used in assays of transient transcriptional activity. **(C)** Activation of *SmTAT1* and *SmPAL1* promoters by SmMYC2 is repressed by SmbHLH37. Effector SmMYC2, alone or together with SmbHLH37, was co-transformed with reporters *P_TAT1_-LUC*, *P_PAL1_-LUC*, and *P_CY P98A14_-LUC*. All data are means of 3 biological replicates, with error bars indicating SD; One-way ANOVA (followed by a Turkey comparison) tested for significant differences among the means (indicated by different letters at *p* < 0.05).

We have reported that overexpression of *SmMYC2* strongly increases the production of RA and Sal B, and those transcript levels of *SmTAT1* and *SmPAL1* are dramatically improved in *SmMYC2*-OE lines (Yang et al., 2017). However, the molecular mechanism had not yet been characterized. Here, we performed Y1H assays and examined transient transcriptional activity to verify whether SmMYC2 directly binds to the promoter regions of these genes to activate their expression. Results from our Y1H assay showed that *Sm*MYC2 did bind to the promoter regions of *SmTAT1*, *SmPAL1*, and *SmCYP98A14* (Figure 7A).

To conduct transient transcriptional activity analysis in *N. benthamiana*, SmMYC2 was used as an effector (Figure 7B). SmMYC2 activated LUC expression under promoters of *SmTAT1*, *SmPAL1*, and *SmCYP98A14*, based on our data from the assay of transient transcriptional activity (Figure 7C). We also learned that SmbHLH37 can repress SmMYC2-activated LUC expression, as driven by the promoters of *SmTAT1* and *SmPAL1* (Figure 7C). Together, these findings suggested that SmbHLH37 antagonizes transcription activator SmMYC2 in the Sal B biosynthesis pathway.

## Discussion

Jasmonates are widely distributed in the plant kingdom (Browse, 2009). They are derived from a-linolenic acid and the biosynthetic enzymes consist of LOX, AOS, AOC, and OPR (Wasternack, 2007). The JAM1/2/3, members of the bHLHs IIId subfamily in *A. thaliana*, have redundant functions that negatively regulate the JA metabolic pathway (Nakata et al., 2013; Nakata and Ohme-Takagi, 2013). We previously reported that SmbHLH37 is most closely associated with AtJAM3 and belongs to the IIId subfamily (Su et al., 2017). Here, overexpression of *SmbHLH37* significantly decreased the level of endogenous JA by repressing the transcripts of *LOX*, *AOC*, *AOS*, and *OPR3*. This indicated that SmbHLH37 is involved in regulation of JA biosynthesis in *S. miltiorrhiza*.

Application of exogenous MeJA is an effective way to improve the yields of secondary metabolites. Earlier research showed that JA signaling has a role in the biosynthesis of salvianolic acids and tanshinones (Xiao et al., 2009; Zhang S. et al., 2011; Pei et al., 2018). Expression of genes in the salvianolic acid and tanshinone biosynthetic pathways is increased significantly after MeJA treatment (Ge et al., 2015; Pei et al., 2018). Our results also indicated that overexpression of *SmbHLH37* significantly decreased RA and Sal B concentrations. Such accumulation profiles were consistent with the expression profiles of all the tested genes involved in Sal B biosynthesis. We previously proposed that *SmbHLH37* helps modulate tanshinone biosynthesis because it is up-regulated by MeJA treatment and is more highly expressed in the roots than in any other organs (Zhang et al., 2015). We also detected tanshinone IIA and cryptotanshinone but found no significant differences in amounts between control plants and *SmbHLH37*-OE lines (data not shown).

Activation of JA signaling can also improve the accumulation of anthocyanin in *S. miltiorrhiza* (Ge et al., 2015). Here, overexpression of *SmbHLH37* significantly decreased the levels of anthocyanin as well as the expression of genes in its biosynthetic pathway. One gene, *DFR*, has a vital role in anthocyanin production (Lim et al., 2016), and we noted that it had the greatest fold-change among the five genes tested here. Therefore, overexpression of *SmbHLH37* repressed overall the biosynthetic pathways for JA, anthocyanin, and salvianolic acids, which is contrary to the activation of JA signaling.

MYC2 is a core TF in the plant response to JAs, inducing JA-mediated responses such as wounding, inhibition of root growth, JA and anthocyanin biosynthesis, and adaptations to oxidative stress (Dombrecht et al., 2007). The JAZ proteins directly interact with MYC2 and inhibit its activity, meaning that they function as repressors of the JA pathway (Chini et al., 2007; Thines et al., 2007; Seo et al., 2011; Song et al., 2011). In *S. miltiorrhiza*, the SmJAZs have proven to be negative regulators of salvianolic acid and tanshinone biosynthesis (Ge et al., 2015; Shi et al., 2016; Pei et al., 2018). In contrast, the orthologs of MYC2 act as positive regulators (Zhou et al., 2016; Yang et al., 2017). Although overexpression of *SmMYC2* increases the production of phenolic acids in *S. miltiorrhiza* (Yang et al., 2017), the responsible molecular mechanism is still unclear.

The bHLH TFs function by binding to the E/G box of the target gene promoters (Shoji and Hashimoto, 2011). Transcriptomic and RT-qPCR analyses of *SmMYC2*-OE and control plants of *S. miltiorrhiza* have shown that transcript levels for *SmPAL1* and *SmTAT1* are increased by 367.1-fold and 110-fold, respectively, in the transgenics (Yang et al., 2017). Both genes contain the E/G-box sequences in their promoters. Our Y1H and transient transcriptional activity assays with tobacco leaves also demonstrated that SmMYC2 directly binds to the promoters of *SmPAL1* and *SmTAT1* to activate their expression. Previous electrophoretic mobility shift assays have shown that SmMYC2a and SmMYC2b bind with the E-box within the *SmCYP98A14* promoter *in vitro* (Zhou et al., 2016). We also confirmed here that SmMYC2 up-regulates the expression of *SmCYP98A14* by binding to its promoter in yeast. Our analysis indicated that the sequence of *SmMYC2a* is almost completely consistent with that of *SmMYC2*. Therefore, we speculate that they are the same gene.

It was documented in Arabidopsis that the bHLH subgroup IIId TFs, including AtJAM1/2/3, negatively regulate JA responses and function as transcription repressors to antagonize the transcription activator MYC2 (Sasaki-Sekimoto et al., 2013; Song et al., 2013; Qi et al., 2015b). We previously reported that overexpression of *SmMYC2* increases the production of phenolic acids in S. miltiorrhiza (Yang et al., 2017). Here, our results showed that SmbHLH37 is most similar to AtJAM3 and overexpression of *SmbHLH37* dampens all analyzed JA responses, that includes the phenolic acids SalB and RA. Antagonistic function between the bHLH subgroup IIId member and MYC2 appears to be a more general mechanism of balance output of the JA pathway.

In *Arabidopsis*, JAM1/2/3 function as transcription repressors to antagonize the transcription activator MYC2 by binding to its target sequences (Song et al., 2013; Qi et al., 2015b). Our results showed that SmbHLH37 employs antagonistic regulation with SmMYC2 by binding to the promoters of the same target genes. The present study indicated that SmbHLH37 directly regulates the Sal B biosynthesis by binding to their promoters of *SmPAL1* and *SmTAT1*. However, the mechanism that OE SmbHLH37 lowered JA and anthocyanin biosynthesis in *S. miltiorrhiza* is unclear. We analyzed the promoter sequences of genes encoding enzymes on the pathways for JA and anthocyanin biosynthesis and found that most promoter sequences contain E/G box (Supplementary Table 3). We speculate that SmbHLH37 probably represses JA and anthocyanin biosynthesis by binding to the promoter regions of the biosynthetic genes. Lowered JA level further affects the accumulation of anthocyanin and phenolic acids. SmbHLH37 negatively impacts on production of anthocyanin and phenolic acids is due to a dual effect, both by a negative feedback loop on JA accumulation, and by repressive binding to promoters of biosynthetic genes.

**FIGURE 8:**
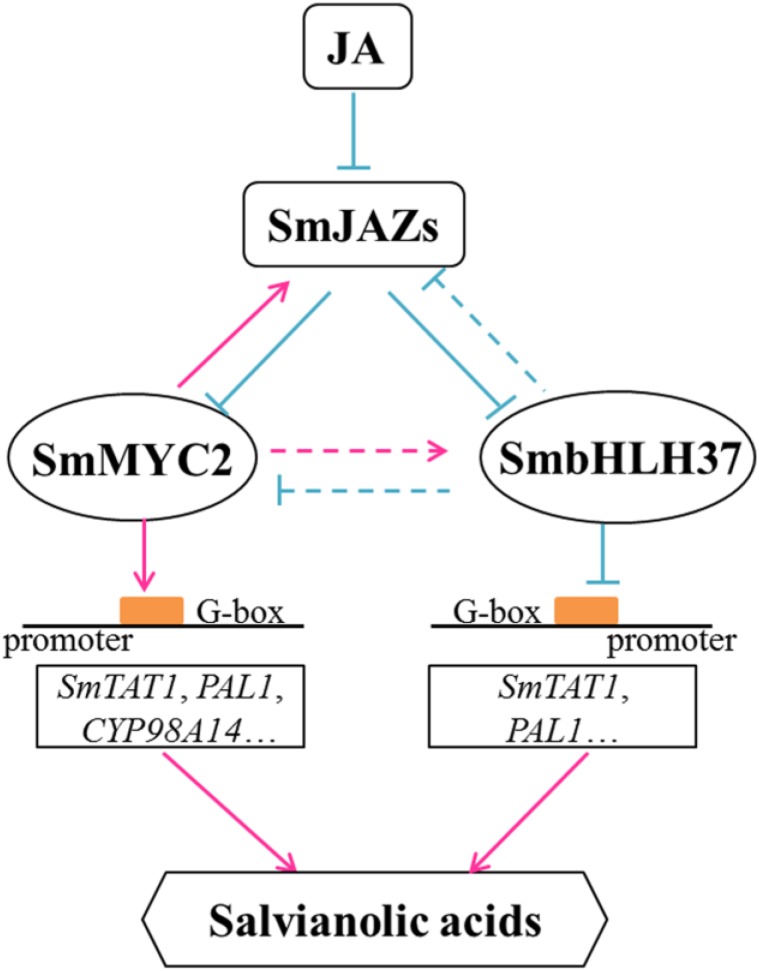
Model illustrating regulation of salvianolic acid biosynthesis by SmbHLH37. Upon perception of JA, JAZ proteins are targeted for degradation. SmbHLH37 and SmMYC2 are then released to regulate, antagonistically or coordinately, their target genes (e.g., *SmTAT1* and *SmPAL1*), which further modulates accumulation of salvianolic acids. SmbHLH37 acts as transcription repressor of JA signaling in *Salvia miltiorrhiza*.

Based on our results and previous reports, we propose a model to illustrate the JA-induced accumulation of salvianolic acids (Figure 8). In it, we confirm that SmbHLH37 regulates such accumulations in *S. miltiorrhiza* by engineering the biosynthetic pathway genes. In the present study, BiFC and Y2H assays indicated that SmbHLH37 interacts with SmJAZ3/8 (Figure 1). We speculate that JAZ proteins regulate SmbHLH37 through protein-protein interactions in a manner similar to the repressive effects of JAZ proteins on the transcriptional activity of MYC2. Jasmonate induces the degradation of JAZ proteins, thereby releasing SmMYC2 and SmbHLH37. The former binds to and activates the promoters of genes involved in salvianolic acid biosynthesis (e.g., *SmTAT1*, *SmPAL1*, and *SmCYP98A14*), ultimately promoting the accumulation of those salvianolic acids. Meanwhile, SmbHLH37 represses these genes and antagonizes this accumulation that is activated by SmMYC2. Both *SmbHLH37* and *SmJAZ*s are more highly expressed in *SmMYC2*-OE lines than in the control (Su et al., 2017; Yang et al., 2017). In contrast, we found here that expression of *SmMYC2* and *SmJAZ*s was lower in *SmbHLH37*-OE lines than in the WT. These data suggest that SmMYC2 activates SmJAZs and SmbHLH37, while SmbHLH37 suppresses SmMYC2 and SmJAZs. Further research is needed on the relationships among SmJAZs, SmMYC2, and SmbHLH37. We speculate that over-expressing SmMYC2 and silencing SmbHLH37 simultaneously is a promising genetic engineering strategy to dramatically enhance concentrations of salvianolic acids.

## Author Contributions

TD performed the experiments and wrote the manuscript. JN performed the experiments and analyzed the data. JS and SL performed the experiments. XG and LL analyzed the data. XC and JK designed the research and wrote the manuscript. All authors read and approved the manuscript.

## Conflict of Interest Statement

The authors declare that the research was conducted in the absence of any commercial or financial relationships that could be construed as a potential conflict of interest.
