# Supplementary material for: SmbHLH37 Functions Antagonistically With SmMYC2 in Regulating Jasmonate-Mediated Biosynthesis of Phenolic Acids in Salvia miltiorrhiza
Source: Front Plant Sci. 2018 Nov 22;9:1720. doi: 10.3389/fpls.2018.01720 (PMC6262058; doi:10.3389/fpls.2018.01720)
Supplement: Supplementary file 1 [file Table_1.docx]

***Supplementary material***

**SmbHLH37 Functions Antagonistically with SmMYC2 in Regulating Jasmonate-mediated Biosynthesis of Phenolic Acids in *Salvia miltiorrhiza***

**Tang-Zhi Du#, Jun-Feng Niu#, Jiao Su, Sha-Sha Li, Xiao-Rong Guo, Lin Li，Xiao-Yan Cao*, Jie-Fang Kang***

^#^These authors contributed equally to this work.

*** Correspondence:** Corresponding Author:

Xiaoyan Cao: [caoxiaoyan@snnu.edu.cn](mailto:caoxiaoyan@snnu.edu.cn).

Jiefang Kang: [kangjiefang@snnu.edu.cn](mailto:kangjiefang@snnu.edu.cn).

**
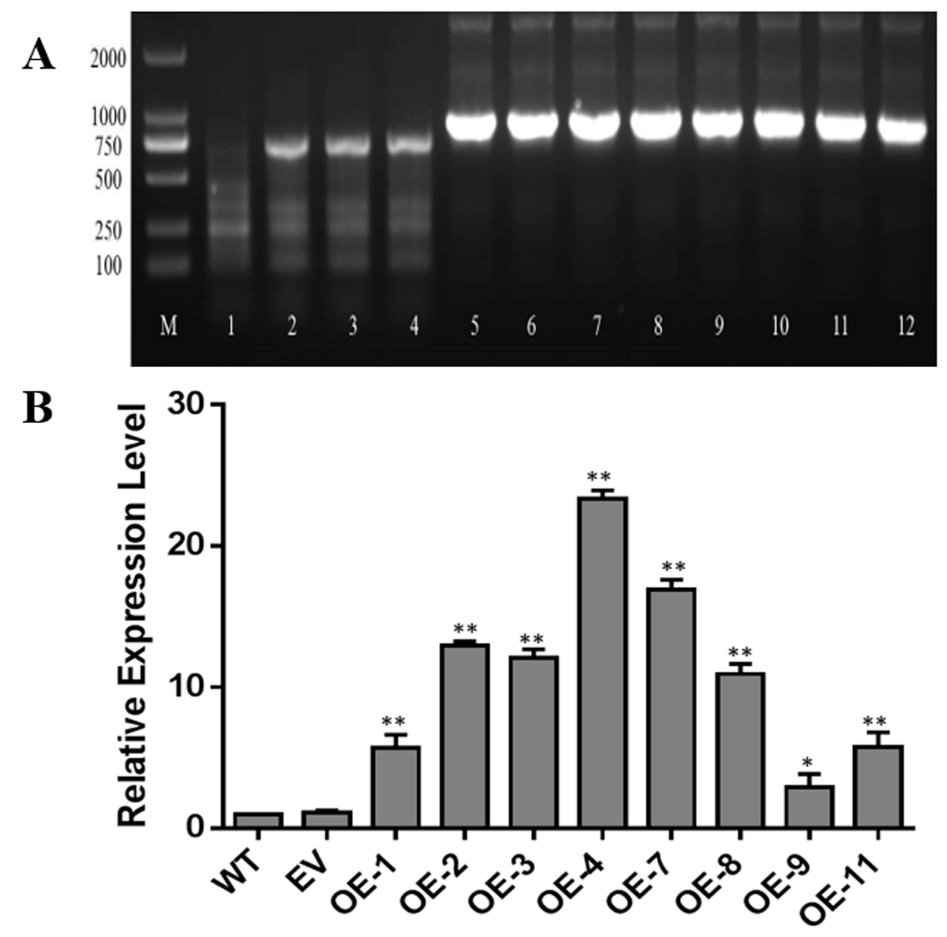
**

**Supplementary Figure 1** Detection of *SmbHLH37*-OE transgenic *Salvia miltiorrhiza* lines. **(A)** PCR detection. M, DL2000 DNA marker; band sizes (bp) are shown on right side. Lanes 1, WT; 2-4, empty vector; 5-12, different transgenic lines. **(B)** Relative expression level of *SmbHLH37* in *S. miltiorrhiza* by RT-qPCR analysis. All data are means of three replicates, with error bars indicating SD; ∗ and ∗∗, values are significantly different from WT at P < 0.05 and P < 0.01, respectively, by the Student’s t-test.


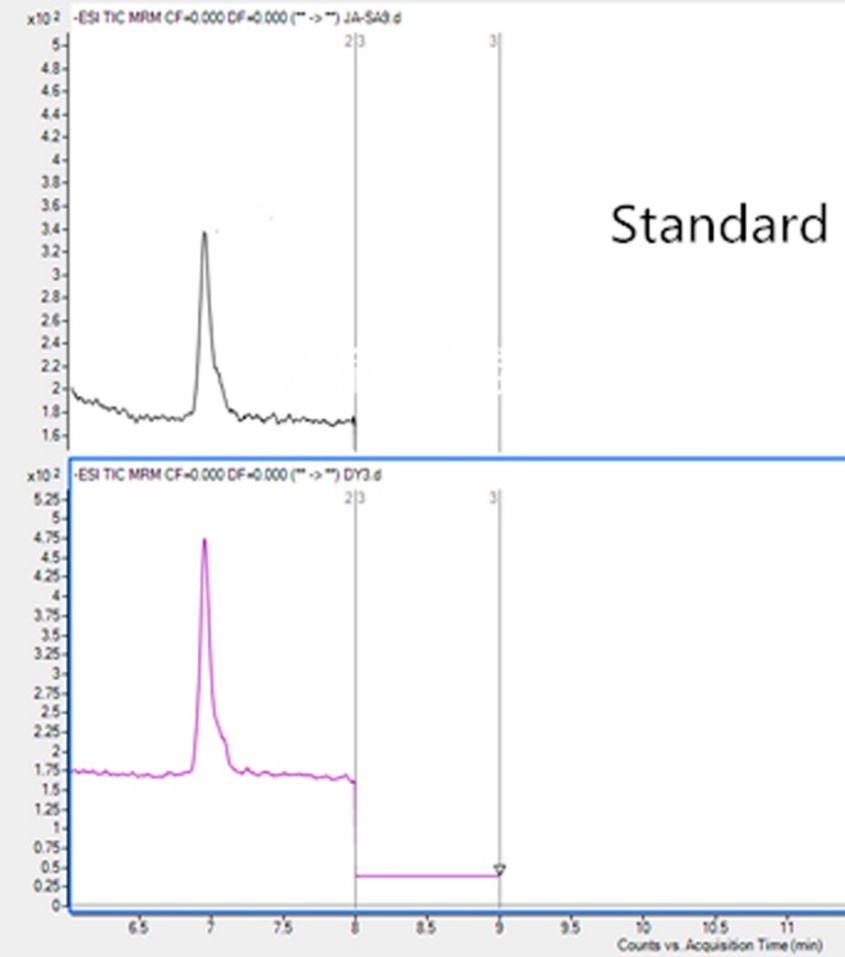


**Supplementary Figure 2** MRM maps of JA standard and samples

The precursor/product ion of JA was 209.1>59.1


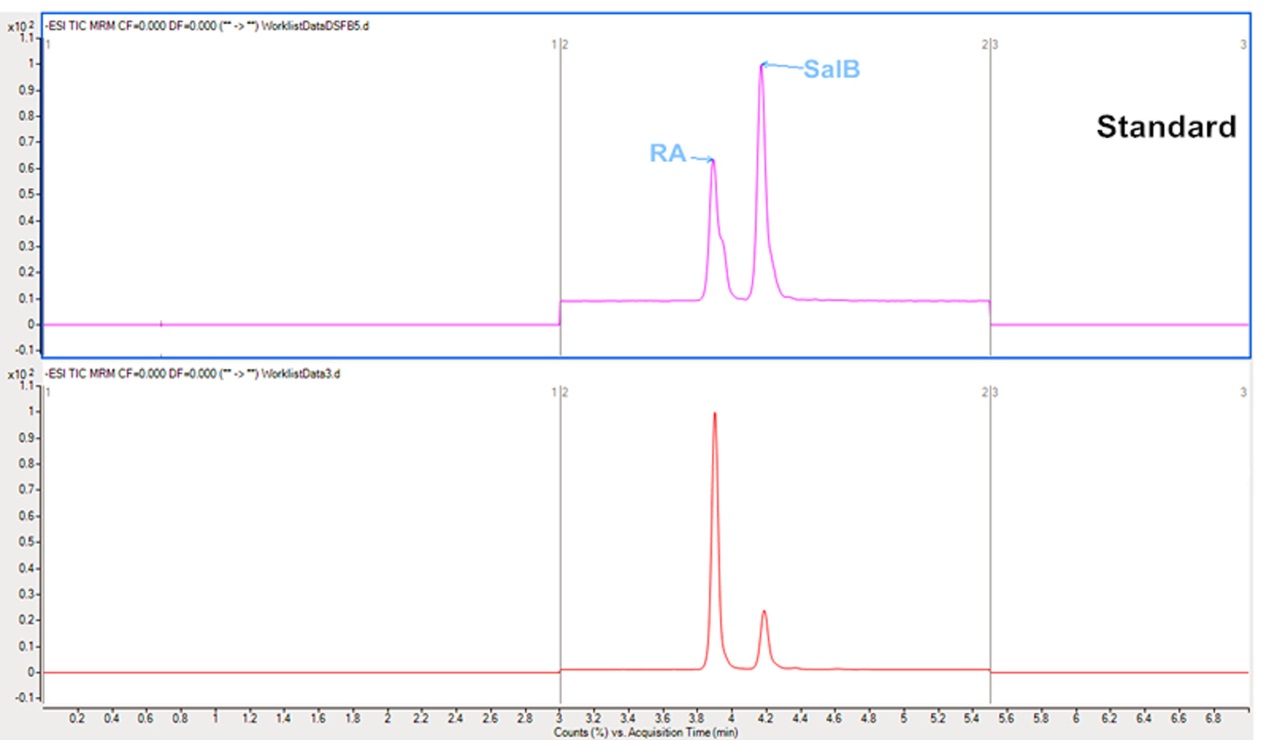


**Supplementary Figure 3** MRM maps of RA and Sal B standard and samples

The precursor/product ion of Sal B and RA was 717.2>519.2 and 359.1>161, respectively.

**Supplementary Table 1.** Primers Used for Vector Construction.

| Primer name | Sequence 5' to 3' |
| --- | --- |
| bHLH37-gene clone F | ATGGGAAAGAAAGTATGGTG |
| bHLH37-gene clone R | TCATTTCAAGAGAGCAGCAG |
| GVbHLH37-688/685/AD/BD/YN/YC F | GGGGACAAGTTTGTACAAAAAAGCAGGCTTC  ATGGGAAAGAAAGTATGGTG |
| GVbHLH37-688/AD/BD R | GGGGACCACTTTGTACAAGAAAGCTGGGTC  TCATTTCAAGAGAGCAGCAGTTA |
| GVbHLH37-/YN/YC R | GGGGACCACTTTGTACAAGAAAGCTGGGTC  TTTCAAGAGAGCAGCAGTTA |
| GVJAZ1-AD/YC F | GGGGACAAGTTTGTACAAAAAAGCAGGCTTC  ATGGTTTCGCCGGAAAAAG |
| GVJAZ1-AD/YC R | GGGGACCACTTTGTACAAGAAAGCTGGGTC  ATGGCGCTGAATTTGGAG |
| GVJAZ3-AD/YC F | GGGGACAAGTTTGTACAAAAAAGCAGGCTTC  ATGGGTTTGTCTGTGAAGCAGGAGG |
| GVJAZ3-AD/YC R | GGGGACCACTTTGTACAAGAAAGCTGGGTC  ATTGGCAGCTGGAACTGGACA |
| GVJAZ8-AD/YC F | GGGGACAAGTTTGTACAAAAAAGCAGGCTTC  ATGAAGCGCAACTGCAATTTG |
| GVJAZ8-AD/YC R | GGGGACCACTTTGTACAAGAAAGCTGGGTC  CTGTCTGGTAATCAGGTATGGCG |
| GVMYC2-AD/YC F | GGGGACAAGTTTGTACAAAAAAGCAGGCTTC  ATGGAATGATTGATTACCGCAC |
| GVMYC2-AD/YC R | GGGGACCACTTTGTACAAGAAAGCTGGGTC  GGAGGAAACTAGACACAAAATACACC |
| bHLH37- pGreenII 62-SK F | CGGGATCCATGGGAAAGAAAGTATGGTG |
| bHLH37- pGreenII 62-SK R | GGGGTACCTCATTTCAAGAGAGCAGCAG |
| MYC2- pGreenII 62-SK F | CGGGATCCATGGAATGATTGATTACCGCAC |
| MYC2-pGreenII 62-SK R | GGGGTACCGGAGGAAACTAGACACAAAATACACC |
| TAT1pro-pGreenII 0800-LUC F | GGGGTACCTACGGAGCCGCCCCTAAAA |
| TAT1pro-pGreenII 0800-LUC R | CGGGATCCGCCGCAACTGAATCGAGAG |
| PAL1pro-pGreenII 0800-LUC F | GGGGTACCCTCGTCCTTGTTTTGCCACT |
| PAL1pro-pGreenII 0800-LUC R | CGGGATCCCACGTTTGCGTTGGTTTAGA |
| CYPpro-pGreenII 0800-LUC F | GGGGTACCTTATTGAACTGGGGGTGAGC |
| CYPpro-pGreenII 0800-LUC R | CGGGATCCGAGCAGGTGGTGAAGGGTAG |
| bHLH37- pGADT7-Rec2 F | CGGAATTCATGGGAAAGAAAGTATGGTG |
| bHLH37- pGADT7-Rec2 R | CGGGATCCTCATTTCAAGAGAGCAGCAG |
| MYC2- pGADT7-Rec2 F | CGGAATTCATGGAATGATTGATTACCGCAC |
| MYC2- pGADT7-Rec2 R | CGGGATCCGGAGGAAACTAGACACAAAATACACC |
| TAT1pro- pHIS2 F | CGAGCTCTACGGAGCCGCCCCTAAAA |
| TAT1pro- pHIS2 R | CGACGCGTGCCGCAACTGAATCGAGAG |
| PAL1pro- pHIS2 F | CGGAATTCCTCGTCCTTGTTTTGCCACT |
| PAL1pro- pHIS2 R | CGACGCGTCACGTTTGCGTTGGTTTAGA |
| CYPpro- pHIS2 F | CGAGCTCTTATTGAACTGGGGGTGAGC |
| CYPpro- pHIS2 R | CGACGCGTGAGCAGGTGGTGAAGGGTAG |

**Supplementary Table 2.** Primers Used for RT-qPCR

| Ubiquitin-F | ACCCTCACGGGGAAGACCATC |
| --- | --- |
| Ubiquitin-R | ACCACGGAGACGGAGGACAAG |
| RT-bHLH37-F | AAGGCTTGAAGATGCTGTTCTA |
| RT-bHLH37-R | ACCGCTCTCTGTTTGAATGG |
| JC-bHLH37-F | GATGACGCACAATCCCACTAT |
| JC-bHLH37-R | AACCTTCACGAGCTCCAAACT |
| 35S-F | ACAAAGGCGGCAACAAACG |
| 35S-R | GCCAGTCTTCACGGCGAGT |
| RT-JAZ1-F | AGGTCCAACTTCTCGCAAAC |
| RT-JAZ1-R | ATCGTCATCTGCCCAATTTC |
| RT-JAZ3-F | CCGTTGGAACCACTGATTTT |
| RT-JAZ3-R | AATGTGCATTTCCAGCCAAT |
| RT-JAZ8-F | GTAACGCCTTCCGTCTCCTT |
| RT-JAZ8-R | TTCCTGCTCGATTTTTCCTC |
| RT-MYC2-F | GAGAAGCGATGTAGTTGTGG |
| RT-MYC2-R | GGTTCACGACTCTGCTACT |
| RT-TAT1-F | CAACTGCTGGTCTTCCACAAAC |
| RT-TAT1-R | GCGAGCCAAAACGGACA |
| RT-TAT3-F | TGCTGAAACTGCCAAGAGGCT |
| RT-TAT3-R | CCGGGCACCAACCATCTCTT |
| RT-PAL1-F | GATAGCGGAGTGCAGGTCGTAC |
| RT-PAL1-R | CGAACTAGCAGATTGGCAGAGG |
| RT-PAL3-F | CTCCACCCGTCGAGGTTCTG |
| RT-PAL3-R | TCTGCATGAGCGGGTACGTG |
| RT-HPPR1-F | TGACTCCAGAAACAACCCACATT |
| RT-HPPR1-R | CCCAGACGACCCTCCACAAG |
| RT-C4H1-F | CCAGGAGTCCAAATAACAGAGCCG |
| RT-C4H1-R | GCCACCAAGCGTTCACCAAGAT |
| RT-4CL2-F | GCGGCGTAGTGCTTCACCTTT |
| RT-4CL2-R | TCGCCAAATACGACCTTTCC |
| RT-4CL3-F | GTCGACGGCGACAACCCTAA |
| RT-4CL3-R | AGCAGCGAGCCGATCTCAAA |
| RT-RAS1-F | CCAAAGTCAATTATGCCAAGGG |
| RT-RAS1-R | GTCGGATAGGTGGTGCTCGT |
| RT-RAS6-F | CCCTCCATTTCATCAGCACG |
| RT-RAS6-R | GATTTGTCGGTGTTGGGGAG |
| RT-CYP98A14-F | ACGTGCGTGTTGCTACGAGAC |
| RT-CYP98A14-R | CGTCGCCAGTGCTGCAACTAA |
| RT-LOX-F | TTCTTCTGTTTCACTGCCTCCT |
| RT-LOX-R | TAACATACCGTGTACCTAACTAC |
| RT-AOC-F | AAGCCTTCTTCGGCCTCTGGAGC |
| RT-AOC-R | TCGTAGCGGTCGGCATTCTGGTC |
| RT-AOS-F | CCCACGCATGAAAGTACACCAGA |
| RT-AOS-R | ATCCTTCCCTGAACCCTCACTCC |
| RT-OPR3-F | TTGAAGCAGGATTTGATGGCA |
| RT-OPR3-R | GGCGAGTTTGGAACCACAGG |
| RT-CHS-F | CGCGATTATGCTTGAGGTTGA |
| RT-CHS-R | CACTACTTGATGTCCCATTTCTTGAC |
| RT-F3’H-F | TCAGGCTTTGAGCAATGGGAAGT |
| RT-F3’H-R | TGAGTCGCATGGGCAGAGGAA |
| RT-F3’5’H-F | CATCTACTCCAACATCGGACAGC |
| RT-F3’5’H-R | CCCCACATAAGGTTCATCAACAG |
| RT-FLS-F | GTTCGTGCATCCCGAGTTCAA |
| RT-FLS-R | CTTCTCGTCACCTCTCGACATCTTT |
| RT-DFR-F | CTCACTACTCCATCATAAAGCAAGG |
| RT-DFR-R | AGTAGAAAACGGCAGCAATCCT |

**Supplementary Table 3.** E/G box identified in the promoter regions of genes encoding for JA and anthocyanin biosynthesis

| Gene (length of promoter upstream ATG) | E/G-box (position upstream ATG) |
| --- | --- |
| *LOX* | CACGTT（-1393） |
| *AOC* | CACGAC（-724）；CACGAC（-694）；CACGAC（-614）；TACGTG（-208）；  **CACGTG**（-163） |
| *AOS* | CACGTT（-348）；CACGAC（-111） |
| *OPR3* | CACGAC（-666） |
| *CHS* | CACGTT（-1353）；TACGTG（-1200）；**CACGTG**（-234）； **CACGTG**（-159） |
| *F3’H* | CACGTT（-1034）；CACGTC（-1027）；CACGTT（-10） |
| *F3’5’H* | CACGTC(-376)；CACGTT（-151） |
| *FLS* | CACGTT（-1332）；CACGTC（-1263）；CACGTC（-915）；CACGTT（-648） |
| *DFR* | TACGTG（-294）；**CACGTG**（-119） |
